# HCV genotype-specific correlation with serum markers: Higher predictability for genotype 4a

**DOI:** 10.1186/1743-422X-8-293

**Published:** 2011-06-10

**Authors:** Waqar Ahmad, Bushra Ijaz, Fouzia T Javed, Humera Kausar, Muhammad T Sarwar, Sana Gull, Sultan Asad, Imran Shahid, Sajida Hassan

**Affiliations:** 1 Applied and Functional Genomics Lab, Centre of Excellence in Molecular Biology, University of the Punjab, Lahore-53700, Pakistan; 2 Fouzia Tahir Javed, Department of Pathology, Jinnah Hospital, Lahore-54590, Pakistan

## Abstract

**Background:**

Several factors have been proposed to assess the clinical outcome of HCV infection. The correlation of HCV genotypes to possible serum markers in clinical prediction is still controversial. The main objective of this study was to determine the existence of any correlation between HCV genotypes to viral load and different clinical serum markers.

**Methods:**

We performed a prospective cross-sectional and observational study. About 3160 serum HCV RNA positive patients were chosen from 4020 randomly selected anti-HCV positive patients. Statistical analysis was performed using the SPSS 16 software package. ROC (receiver operating characteristics) curves were used to compare diagnostic values of serum markers to predict genotypes.

**Results:**

The most prevalent genotype was 3a (73.9%) followed by 1a (10.7%), 4a (6.4%) and 3b (6.1%) in Pakistani population. No correlation was found between viral load and serum markers for genotype 3a in a large no. of sample (n = 2336). While significant correlation was observed between viral load and AST in genotype 3b, ALP with viral load and ALT for genotype 1a. Patients with genotype 4a showed a significant inverse correlation with viral load and Hb level and AST with ALP. For genotype 4a, AUC (area under the curve) of ALT, ALP, AST, bilirubin, Hb level and viral load was 0.790, 0.763, 0.454, 0.664, 0.458 and 0.872 respectively.

**Conclusions:**

In conclusion, there was a significant variable response of HCV genotypes with serum markers. Severity of disease is independent of serum marker level in genotype 3a, while the liver damage in genotype 4a may associate with viral cytopathic effect as well as the immune-mediated process. An index using six serum markers may correctly predict genotype 4a in patients with ≥75% accuracy.

## Introduction

Hepatitis C virus (HCV) is a major cause of liver associated diseases all over the world. An estimated 3% of the world's populations (more than 350 million people) are chronically infected with HCV, which is the main cause of liver fibrosis, cirrhosis and hepatocellular carcinoma (HCC) in a substantial number of patients [1,2]. Due to considerable sequence diversity and sequence comparisons in different parts of hepatitis C virus genome, classification of the virus into a series of genotypes showed distinct geographical and frequency distribution across the whole world [3-6].

Approximately, 10 million people in Pakistan are infected with HCV [7]. It is well established factor that in patients infected with HCV, the clinical findings, genotypes and viral load are strong predictors for the outcome of antiviral therapy [8,9]. The most prevalent genotype in Pakistan is 3a followed by 3b and 1a [10]. Due to high prevalence of genotype 3a in Pakistan; HCV genotyping is not recommended for HCV infected patients routinely by Pakistan's Society of Gastroenterology [11]. Secondly, due to poverty and cost of genotyping test, many patients refused to do genotyping. Nevertheless, genotyping is important because it not only provides information as to strain variation and potential association with disease severity but also related to the possibility of treatment response, as the treatment plan of chronic HCV infection with interferon varies with the genotype being treated [12,13]. It is reported that treatment with interferon is more effective in patients with genotypes 2 and 3 than in patients infected with genotypes 1 and 4 [14,15]. Several studies revealed that HCC develops in 1-4% of patients and liver biopsy is considered the gold standard to identify liver fibrosis. Unfortunately, procedure of liver biopsy is invasive, expensive and unsuitable for all patients with severe side effects leading to death [1,11,15,16]. An assessment of the disease development based on clinical findings is still critical for patients infected with HCV. At present, the clinicopathological significance of serum biochemical markers and viral load and their relationship among different genotypes is not well known. Several authors tried to find accurate noninvasive markers of liver damage and developed correlations between the serum hyaluronic acid levels, collagen level, platelet count, serum bilirubin levels, HCV viral load, genotypes and elevated ALT/AST levels in HCV infected patients, but no clear conclusions were formed [17-23].

In present study, we investigated the correlation of several clinical findings like Hb level, bilirubin level, ALT, ALP and AST and viral load in patients with different genotypes. The ideal serum markers for genotype determination should have good sensitivity, be readily available, inexpensive, reproducible, safe and able to predict genotypes with accuracy. The need of genotyping may be eradicated if the serum biochemical markers with high positive or negative predictive values of several genotypes can be obtained and thus minimize the cost of genotyping and liver biopsy.

## Materials and methods

### Patients

Patients of this study were the people referred to Pathology department, Jinnah Hospital, Lahore, Pakistan, for biochemical and serological tests. This analytical study was carried out from March 2007 to September 2009 with collaboration of National Centre of Excellence in Molecular Biology, University of the Punjab, Lahore, Pakistan. Blood samples (10 mL) were collected from each patient and tested for anti-HCV antibody by ELISA. Adult (≥18 years) patients with positive serology and/or positive test for HCV alone and no evidence of liver failure were included in this study. Patients who were not keen to give informed consent, not able to make follow-up visits and not willing to undergo genetic testing and allow samples to be stored for future research were excluded from the study. Accordingly, thus, 3160 HCV-RNA positive patients from 4020 HCV antibody (anti-HCV)-positive persons were identified. Questioner (including their personal, lab tests and demographical information, possible transmission route of HCV infection, clinical, virological and biochemical data) was prepared for patients who came for HCV initial screening and further genotyping and viral load quantification. The routine liver function tests (LFTs), Hb level and direct bilirubin were estimated for each patient in the hospital laboratory. Informed consent was obtained from patients. The study was approved by the institutional ethical committee.

### HCV antibody and viral assays

HCV detection and genotyping was performed at the Department of Pathology, Jinnah Hospital, Lahore, Pakistan. RNA was extracted from 140µl of serum samples using QIAamp viral RNA extraction kit (Qiagen, USA) according to the manufacturer's protocol. cDNA was synthesized using Moloney murine leukemia virus (MmLV) followed by PCR using primers derived from the 5'UTR non-coding region of HCV genome described by Chan *et al. *[24]. For HCV RNA quantification, Qiagen HCV RG RT-PCR assay was used. Quantification was carried out with 10 ul of the extracted RNA on Rotor-gene Real-Time PCR machine (USA) using fluorescent probes to detect amplification after each replicating cycle as described by manufacturer protocol. The lower limit of detection for this assay is 1000 IU/ml HCV and genotyping was carried out using Invader HCV genotyping assay (Third wave technology, USA). Briefly, 100 ng of the HCV RNA was reverse transcribed to cDNA using 200 U of MmLV (Invitrogen, USA). From the amplified product, 2µl were taken and the genotyping assay was performed for 12 different HCV types.

### Statistical analysis

Statistical analysis was performed using the statistical package for social studies (SPSS) version 16 for windows. Student t-test and Chi-square tests were applied to evaluate differences in proportions. *P *value <0.05 was considered significant. Univariate analysis includes the variables age, sex, Hb level, bilirubin, ALT, AST, ALP and viral load. Age, sex and genotypes were taken as independent categorical factors. The normal values of ALT, ALP, AST and direct bilirubin level were (~5-40 IU/ mL), (< 120 IU/ mL), (~10-40 IU/ mL) and (< 0.4 mg/ dl) respectively. Multiple regression analysis was used to evaluate independent associations between HCV genotypes and individual demographic characteristics and biochemical values to identify variables association within different genotypes. Once we determined that differences exist among the means, post hoc range tests and pair-wise multiple comparisons were determined. Comparisons were made on unadjusted values fixed between-patients factors only. The relationship between serum markers and genotypes were analyzed by Spearman's correlation for non-parametric data and by the Pearson method for parametric data. To obtain cutoff values of serum markers for HCV genotypes, receiver operating characteristics (ROC) curves were drawn for serum markers by plotting sensitivity of the assays against false positivity (1-specificity [25]. Comparison of the area under the curve (AUC) was used to assess the overall diagnostic values of serum markers.

## Results

### Prevalence of HCV infection

Of the 4020 ELISA positive patients, 3160 (78.6%) showed positive PCR while 860 (21.4%) were negative for HCV. Out of 3160 patients with positive PCR, 1515 (48%) were males while 1645 (52%) were females. The median age of patients was 37 years (range 18-75). Age of the patients was taken as a continuous as well as a categorical variable. Patients were divided into two age groups i.e. ≤ 40 years and >40 years. In age group ≤ 40 years there were 2119 patient, while age group >40 years include 1041 patients.

### Genotype distribution among patients

Based on weighted analysis of patients infected with HCV, the most frequently detected genotype was 3 (80%), with predominant subtype 3a (73.9%) and 3b (6.1%). Genotype 1 (10.7%) was exclusively consisted of the subtype 1a, while genotype 4 (7.1%) comprised the subtype a (6.4%) and b (0.7%). Patients with mix genotype 4 &5 (0.8%) and untypable genotype 1(0.2%) were also identified. The genotype 2b was detected in only three (0.1%) patients. The frequency distribution of different genotypes according to age groups and gender is given in Table 1. Distribution of genotypes among age groups was not statistically different (*p *= 0.488). Genotype subtype 3a was the most prevalent genotype in both age groups followed by 1a. Only three patients (0.14%) with genotype 2b were observed in age group ≤ 40 years. Overall prevalence of genotypes within gender was also statistically non-significant (*p *= 0.098).

**Table 1:** Genotype-specific representation according to gender and age

	Genotypes							
	
Characteristics	1	2	3		4		Mix	Untypable
	
	1a	2b	3a	3b	4a	4b	4&5	N.T
Total (n = 3160)	339 (10.7%)	3 (0.09%)	2336 (73.9%)	194 (6.1%)	202 (6.4%)	22 (0.69%)	26 (0.82%)	38 (1.2%)
Mean Age (SD)	36.3 (9.63)	36.67 (2.8)	37.39 (10.42)	36.19 (10.62)	37.01 (10.24)	32.45 (9.5)	33.46 (9.7)	35.42 (9.7)
Age Range (years)	18-66	35-40	18-75	18-74	18-70	18-55	18-51	18-50
**Age Groups (years)**								
≤40 (n = 2119)	242	3	1534	140	136	18	21	25
≥ 40 (n = 1041)	97	0	802	54	66	4	5	13
**Sex**								
Male (n = 1515)	152	0	1117	111	95	11	9	20
Female (n = 1645)	187	3	1219	83	107	11	17	18

### Association of age, gender and genotypes with serum markers and viral load

**Figure 1:**
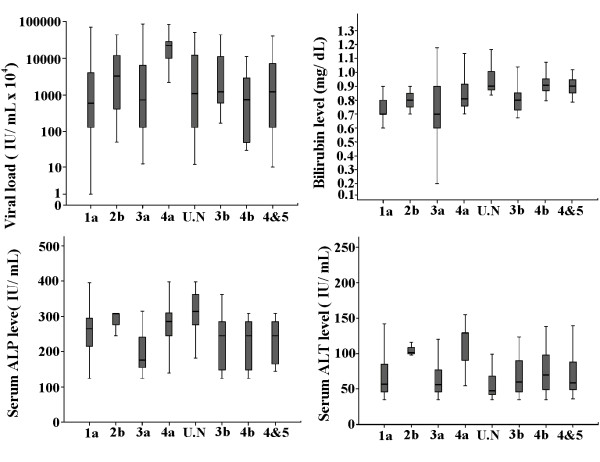
**Variation of significant serum markers among genotypes**. Box plots of four significant serum markers i.e. viral load, bilirubin level, Serum ALP and ALT in relation with eight different HCV genotypes. The line through the middle of the box is the median while the top and bottom of the box are 25^th ^and 75^th ^percentiles.

Univariate analysis (Table 2) revealed that all serum markers were independently distributed between gender and age groups while serum markers including direct bilirubin level, serum ALP and ALT levels and viral load were significantly different among genotypes. Box plots of the above four significant serum markers with eight different HCV genotypes are shown in Figure 1. The overall mean bilirubin value was 0.74 ± 0.18 mg/dL. The bilirubin level was high in genotype 2b (0.80 ± 0.10), 4a (0.81 ± 0.05), 4b (0.90 ± 0.12), 4&5 (0.89 ± 0.003) and untypable (0.88 ± 0.09) while genotype 1a (0.706 ± 0.14) and 3a (0.735 ± 0.20) showed a low bilirubin level. Serum ALP levels (mean 209.03 ± 64 IU/mL) were higher in genotype 1a (251 ± 62.2), 2b (287 ± 36.37), 3b (221.1 ± 70.1), 4a (275.7 ± 70.5), 4b (227.2 ± 72.1), 4&5 (232.7 ± 63.1) and untypable (315.1 ± 54.1) in comparison to genotype 3a (193.9 ± 54.3). Serum levels of ALT (mean value 71.1 ± 35.06 IU/mL) were significantly elevated in 2b (105 ± 9.64) and 4a (108.1 ± 35.6) genotypes as compared to 1a (72.1 ± 36.5), 3a (67.6 ± 32.9), 3b (72.6 ± 35.6), 4b (75.6 ± 31.3) and mix genotype 4&5 (74.4 ± 37.02), while serum ALT levels were significantly low in patients with untypable genotype (57.65 ± 23.5). Viral load varied from 1.1 × 10^3 ^IU/mL to 8.5 × 10^8 ^IU/mL (mean, 6.8 × 10^7 ^± 1.2 × 10^8^). The viral load was significantly higher in genotype 2b (1.5 × 10^7 ^± 2.3 × 10^7^) and 4a (2.1 × 10^7 ^± 1.1 × 10^7^) while the patients with genotype 1a (4.5 × 10^6 ^± 9.8 × 10^6^), 3a (5.9 × 10^6 ^± 1.1 × 10^7^), 3b (7.1 × 10^6 ^± 1.1 × 10^7^), 4b (1.9 × 10^6 ^± 3.1 × 10^6^), 4&5 (6.7 × 10^6 ^± 1.1 × 10^7^) and untypeable (8.1 × 10^6 ^± 1.1 × 10^7^) showed intermediate viremia levels. Genotype 2b, 4b, mix and untypable were eliminated from further analysis because of small sample size. Further Multivariate analysis in Table 3 revealed that bilirubin, ALP, ALT levels and viral load were significantly different among genotypes 1a, 3a, 3b and 4a.

**Table 2:** Univariate analysis of patient's data by age, sex and genotype

Factors Studied	Serum Markers	95% Confidence Interval		F-value	*p*-value
				
		Lower Bound	Upper Bound		
**AGE**	Hb level	12.800	12.986	1.759	.134
	Bilirubin level	.735	.753	.308	.873
	ALP	204.201	210.449	1.007	.402
	ALT	68.395	71.810	1.056	.377
	AST	66.299	69.655	1.017	.397
	Viral load	6409.094	7578.840	.698	.593
**GENDER**	Hb level	12.783	12.916	2.537	.111
	Bilirubin level	.739	.752	.164	.686
	ALP	206.795	211.275	.004	.952
	ALT	69.798	72.246	2.380	.123
	AST	66.695	69.101	.730	.393
	Viral load	6425.361	7263.954	.021	.886
**GENOTYPE**	Hb level	12.361	12.996	1.295	.248
	Bilirubin level	.787	.847	17.846	.000
	ALP	240.926	260.033	108.589	.000
	ALT	73.576	84.729	39.728	.000
	AST	63.472	74.892	1.070	.380
	Viral load	6944.945	10731.895	48.110	.000

**Table 3:** Multivariate analysis of significant serum markers among genotypes 1a, 3a, 3b and 4a

Serum Markers	Genotypes	95% Confidence Interval		Hypothesis Test	
		
		Lower	Upper	Wald Chi-Square	**Sig**.
**Bilirubin level**	1a	-.262	-.117	26.314	0.000
	3a	-.231	-.091	20.225	0.000
	4a	-.250	-.099	20.463	0.000
**ALP**	1a	17.962	18.760	8140.621	0.000
	3a	-61.075	-16.561	11.685	0.001
	4a	42.554	43.371	42517.226	0.000
**ALT**	1a	-2.660	-1.862	123.417	0.000
	3a	-7.100	-6.327	1159.056	0.000
	4a	33.247	34.063	26090.882	0.000
**Viral load**	1a	-2221.812	-2221.014	1.192E8	0.000
	3a	-771.541	-770.768	1.529E7	0.000
	3b	454.651	455.470	4.747E6	0.000
	4a	14092.652	14093.469	4.575E9	0.000

### Correlation of Significant Serum Markers and Viral load within HCV genotypes

**Figure 2:**
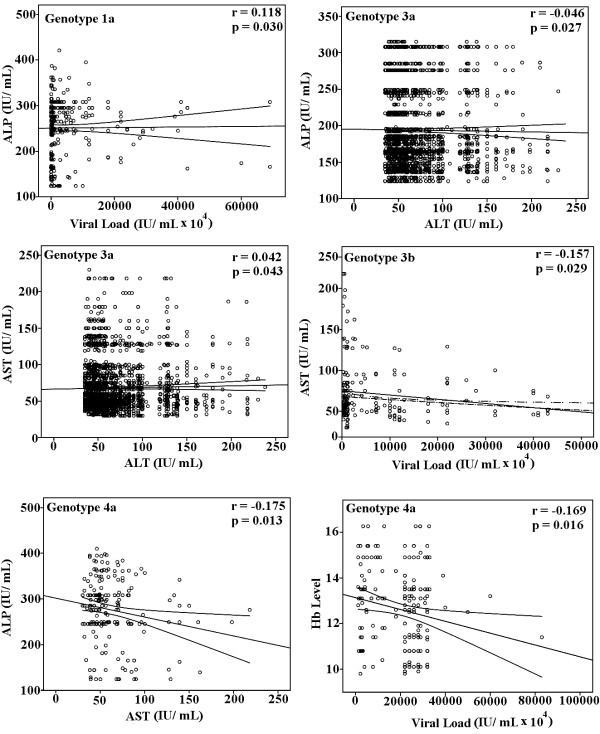
**Correlation of serum markers with each other in HCV genotypes**. Significant correlation between different serum markers in genotypes 1a, 3a, 3b and 4a was found. This can lead to different possible mechanisms of liver injury in different genotypes.

**Figure 3:**
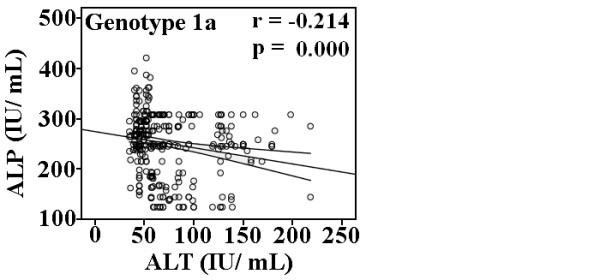
**Association of ALP and ALT in HCV genotype 1a**. A negative significant correlation was observed between ALP and ALT in patients with genotype 1a.

A correlation of significant serum markers with each other and viral load in HCV infected patients is illustrated in Figure 2 showed that viral load has positive correlation with ALP in genotype 1a (*r *= 0.118, *p *= 0.030), linear significant correlation of viral load and AST was found in 3b (*r *= -0.157, *p *= 0.029), while negative linear correlation of Hb level with viral load in genotype 4a, was observed (*r *= -0.169, *p *= 0.016). Regarding relationship between clinical factors, we observed significant correlations between ALP and ALT (*r *= -0.214, *p *= 0.000) in genotype 1a (Figure 3), ALT and AST (*r *= 0.042, *p *= 0.043) and ALP and ALT (*r *= -0.046, *p *= 0.027) in genotype 3a, and AST and ALP (*r *= -0.175, *p *= 0.013) in genotype 4a.

### Determination of cutoff values of serum markers to predict genotypes

**Figure 4:**
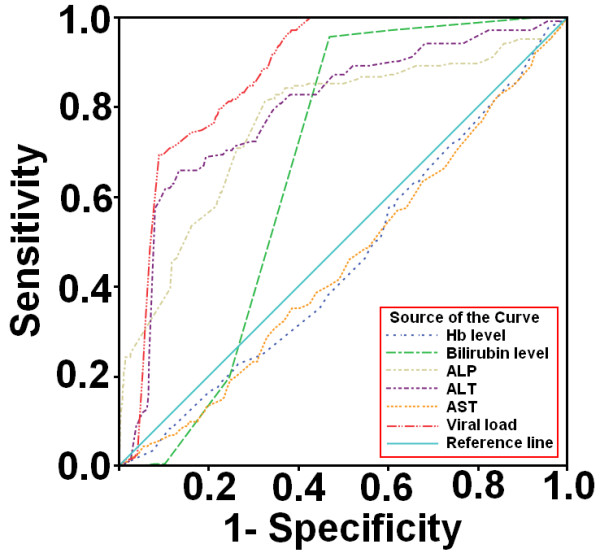
**Receiver operator characteristic (ROC) curves of six serum markers**. ROC curves were drawn to evaluate best cutoff points for predicting genotypes. ROC curves of serum markers in patients showed that viral load, ALP, ALT and bilirubin level can better predict genotype 4a at given cutoff values.

ROC curves (Figure 4) to predict genotypes among patients were plotted against serum markers and we were able to found best cutoff points for genotype 4a. The best cutoff values calculated to predict genotype 4a were as: Hb level ≤ 11.85 g/dL, bilirubin level ≥ 0.75 mg/ dL, ALP level ≥ 243 IU/ mL, ALT≥ 125 IU/ mL, AST level ~ 40-75 IU/ mL and serum viral load ≥ 1 × 10^7 ^IU/mL. The cut off values with significant positive predictive values (PPV) and negative predictive values (NPV) and specificity and sensitivity for genotypes 4a are given in Table 4. ALT, ALP, viral load and bilirubin levels showed high PPV and NPV as compared to AST and Hb level. Moreover, during evaluation the area under the curve (AUC) of each serum marker as illustrated in Table 5 we observed high AUC for ALP 0.763 (95% Cl 0.724-0.801, *p *= 0.000), ALT 0.790 (95% Cl 0.756-0.824, *p *= 0.000) and viral load 0.872 (95% Cl 0.854-0.890, *p *= 0.000) while bilirubin level 0.664 (95% Cl 0.641-0.0.686, *p *= 0.000), AST 0.454 (95% Cl 0.414-0.494, *p *= 0.028) and Hb level 0.458 (95% Cl 0.418-0.497, *p *= 0.045) showed moderate AUC.

**Table 4:** Sensitivity, specificity, and cutoff values of the six serum markers to predict genotype 4a

SERUM MARKERS	Cutoff values	Sensitivity	Specificity	PPV (%)	NPV (%)
**Hb level**	11.85 g/dL	60	37	65	44
**Bilirubin level**	0.75 mg/ dL	96	53	96	60
**ALP**	243 IU/ mL	81	62	82	78
**ALT**	125 IU/ mL	62	89	62	96
**AST**	~ 40-75 IU/ mL	51	45	65	64
**Viral load**	1 × 10^7 ^IU/ mL	75	82	77	86

**Table 5:** AUROC analysis of serum markers for predicting genotype 4a in chronic HCV patients

Serum Marker	Area	Std. Error	Significance	95% Confidence Interval	
				
				Lower Bound	Upper Bound
**Hb level**	0.458	0.020	0.045	0.418	0.497
**Bilirubin level**	0.664	0.011	0.000	0.641	0.686
**ALP**	0.763	0.020	0.000	0.724	0.801
**ALT**	0.790	0.017	0.000	0.756	0.824
**AST**	0.454	0.020	0.028	0.414	0.494
**Viral load**	0.872	0.009	0.000	0.854	0.890

## Discussion

The difference in distribution of HCV genotypes suggests the subsistence of diverse form of disease acquirement. The basic aim of this study was to evaluate the predictive value of a combination of basic serum biochemical markers for the diagnosis of genotypes and their relation to disease outcome.

The present study was conducted in randomly selected samples of general population in Pakistan (Table 1). Our patient's data showed no significant differences in genotype distribution in relation to gender and age groups. Various genotypes, particularly 1, 3 and 4 were equally distributed in gender and age groups. Prevalence of genotype in our study was: genotype 3 (n = 2530, 80%), followed by genotype 1 (n = 339, 10.8%) and genotype 4 (n = 224, 7.08%). Subtypes 3a, 1a and 4a were predominant, whereas mix subtype 4a/5a was also found in some patients (n = 26, 0.8%). Among patients, 1.2% (n = 38) showed untypable genotype.

The correlation among HCV genotypes with viral load and serum markers and their association with disease severity and sensitivity to interferon treatment remains controversial till date [26,27]. Evaluating the correlation between different clinical markers with genotypes, our results showed that a combination of four clinical markers (ALT, AST, ALP and bilirubin level) and serum viral load can have high positive or negative predictive value for diagnosis of different HCV genotypes. Our data showed significant increase in bilirubin levels in patients with genotypes 4, mix (4&5) and untypable. High bilirubin level is usually associated with liver metastases and liver tumor involvement leading to hepatocellular carcinoma and liver cirrhosis by active or non-active HCV or HBV [28]. As different genotypes lead to diverse severity levels of liver disease so the treatment plan of chronic HCV infection with interferon varies with the genotype being treated [29]. Bilirubin may be used as marker of liver injury and to determine the proper dose of interferon in patients with different genotypes. Elevated aminotransferases levels act as indicators of liver cell injury and are usually predominant in liver cirrhosis with increased ALT levels [20,30]. We observed elevated ALT and AST levels in all genotypes compared to normal range but in patients infected with genotype 4a values were quite higher (>2 times to normal range 5-40 IU/ mL). These results could lead to the confirmation association of genotype 4a with increased risk of cirrhosis [31]. In previous studies, serum ALP levels were not considered valuable markers during HCV diagnosis but recent studies revealed that the higher levels of ALP are usually associated with liver metastasis, extraheptic bile obstruction, primary biliary cirrhosis, intraheptic cholestasis, infiltrative liver disease, hepatitis, cirrhosis, primary sclerosisng cholangitis, hepatic lymphoma, liver abscess, sarcoidosis and congestive cardiac failure [31-33]. A change in ALP levels greater than 120 U/L can be indicative of advanced disease progression [34]. In our study patients with genotype 4a reflected high ALP levels as compared to others as illustrated in Figure 1.

Patients infected with genotype 3a showed negative correlation between ALP and ALT while a positive correlation was also found between AST and ALT in patients infected with genotype 3a. No correlation between serum viremia levels and other serum markers in genotype 3a in our study was in agreement with outcome of Azzari et al and Abraham et al that the viral load was independent of ALT activity in HCV [18,35]. A linear relationship established between serum HCV RNA levels and amount of virus in liver or serum HCV RNA levels and liver injury and vice versa in many studies. The involvement of different factors like different clinico-histopathological evaluation procedures [2,16] can lead to the opinion that liver injury in HCV infection due to genotype 3a is not directly associated with serum viremia levels or the number of infected hepatocytes. Zechini et al found a relation between HCV viral load and AST. However, we observed a negative correlation between viral load and AST in genotype 3b that may be due to poor immune response resulting in lower AST level and higher viral load and vise versa lead to liver damage [19,36].

In patients infected due to genotype 1a, viral load showed significant positive correlation with ALP, and ALP with ALT. Serum ALT and ALP can be used for assessing the liver function status in anti-HCV positive patients [37]. As elevated levels of ALP, ALT and AST are associated with liver injury leading to cirrhosis, HCV infection with genotype 1a could lead to more severe liver damage as compared to genotype 3.

In our study patients infected with genotype 4a showed high serum viral loads as compared to others, while an inverse correlation between viral load and Hb level and serum AST and ALP levels in genotype 4a was also observed. Kato et al monitored significantly higher HCV RNA level in patients with chronic active hepatitis and cirrhosis compared to chronic persistent hepatitis [38]. As HCV is associated with many extra hepatic complications involving renal, articular, neuorologic, cutaneous and haemopoietic systems, several autoimmune phenomenon are observed in patients infected with HCV [39,40], decline of Hb level with increase of viral load in genotype 4 may lead to autoimmune haemolytic anemia (AIHA) that can contribute to enhance the liver cirrhosis in genotype 4 as the patients with HCV related AIHA have higher prevalence of cirrhosis [41]. Higher ALP and bilirubin levels and mild increase in AST levels in patients with genotype 4a may lead to cholestatic hepatitis that is a severe form of HCV recurrence after treatment and organ transplantation like liver, kidney and heart [42,43].

Based on our findings, we calculated the optimum cutoff values of serum markers by generating ROC curves to predict HCV genotypes in HCV-RNA positive patients. To assess the diagnostic accuracy of serum markers to evaluate patients require the selection of a decision threshold. As, both sensitivity and specificity are equally important in classifying patients with positive PCR for genotypes, we were able to find the best cutoff values of each serum marker for genotype 4a that can maximize the sum of sensitivity and specificity as illustrated in Table 4. At given cutoff values for each serum marker in patients infected with genotype 4a, 60% have raised ALT ≥ 125 IU/ mL, 82.5% have elevated ALP ≥ 243 IU/ mL, 99.5% have bilirubin level more than ≥ 0.8 mg/ dL and 95% with raised serum viremia levels up to ≥ 1 × 10^7 ^IU/mL, 75.2% have low Hb level (≤ 11.85g/ dL) and 65% showed mild increase in AST levels (40~75 IU/ mL). The percentage accuracy of each serum marker at given cutoff values is; Hb level 58%, bilirubin level 73.2%, ALP 70.3%, ALT 86.5%, AST 44.6% and viral load 84.6%. These all serum markers in combination may predict genotype 4a in patients with more than 75% accuracy.

In Pakistan due to poverty, doctors usually do not recommend the genotype testing, as they consider HCV 3a more prevalent and INF therapy responder, but due to high frequency of genotype 1a and 4a and for better dose administration, time course of INF therapy and sustainable response, importance of genotyping cannot be neglected. It is conceivable that serum viral load, ALT, ALP and bilirubin levels are suitable factors that may determine liver damage as well as HCV genotypes. Although all genotypes showed significant variable response to the serum markers, we were able to find serum markers with viral load that can predict genotype 4a, with more than 75% accuracy. However we recommend genotyping assay to find possible association with disease severity and guide about treatment duration and outcomes. Future studies are required in liver biopsy samples to confirm the association we found in this study.

## Abbreviations

HCV: hepatitis C; PPV: positive predicted value; NPV: negative predicted value; AUC: area under the curve; ROC: receiver operating characteristic

## Competing interests

The authors declare that they have no competing interests.

## Authors' contributions

WA and BI contributed equally to this study. WA, BI and SH designed the study, analyze the data and wrote paper. AS, HK, SG, Sarwar MT and IS performed all lab work. FTJ and SA collected and arranged data. All work was performed under supervision of SH. All the authors read and approved final version of manuscript.
